# Targeted NGS Revealed Pathogenic Mutation in a 13-Year-Old Patient with Homozygous Familial Hypercholesterolemia: A Case Report

**DOI:** 10.3390/ijms252211882

**Published:** 2024-11-05

**Authors:** Ayaulym E. Chamoieva, Zhanel Z. Mirmanova, Madina R. Zhalbinova, Saule E. Rakhimova, Asset Z. Daniyarov, Ulykbek Y. Kairov, Almira I. Baigalkanova, Murat A. Mukarov, Makhabbat S. Bekbossynova, Ainur R. Akilzhanova

**Affiliations:** 1 National Laboratory Astana, Nazarbayev University, Astana 010000, Kazakhstan; 2 Eurasian Society of Personalized Medicine, Astana 010000, Kazakhstan; 3 Faculty of Natural Sciences, L.N. Gumilyev, Eurasian National University, Astana 010008, Kazakhstan; 4 Corporate Fund “University Medical Center”, National Research Cardiac Surgery Center, Astana 010000, Kazakhstan

**Keywords:** familial hypercholesterolemia, homozygous familial hypercholesterolemia, low-density lipoprotein receptor, mutation

## Abstract

Familial hypercholesterolemia is an autosomal hereditary disease defined by an increased level of low-density lipoprotein cholesterol (LDL-C), which predisposes significant risks for premature cardiovascular disorders. We present a family trio study: proband, a 13-year-old Kazakh girl with homozygous familial hypercholesterolemia (HoFH) and her parents. HoFH is much more rare and severe than a heterozygous form of the disorder. HoFH patients generally present with LDL-C levels exceeding 13 mmol/L, resulting in early and life-threatening cardiovascular events within the first decades of life. In cases of neglected treatment, young patients have a risk of death from coronary diseases before the age of 30. The aim of this research was to identify genetic mutations in the affected patient and her parents. Genetic testing was necessary due to highly elevated LDL-C levels and the presence of multiple xanthomas. Targeted next-generation sequencing (NGS) was performed in this study using the Illumina TruSight cardio panel, which targets 174 genes related to cardiac disorders. The girl was diagnosed with HoFH based on the results of genetic testing. A biallelic mutation was observed in exon 3 of the low-density lipoprotein receptor (*LDLR*): c. 295 G>A (p.Glu99Lys). Sanger sequencing confirmed that the mutant gene was inherited from both parents. After confirming the genetic diagnosis of HoFH, the patient was treated with LDL apheresis and statins. This case report is the first study of HoFH in a pediatric patient from the Central Asian region. Globally, it emphasizes the need for increased clinical awareness among healthcare providers, as early detection and intervention are important for improving outcomes, particularly in pediatric patients with this rare genetic disorder.

## 1. Introduction

Familial hypercholesterolemia (FH) is a genetic disorder characterized by a clinical triad of significantly high levels of low-density lipoprotein cholesterol (LDL-C), premature coronary artery disease (CAD), and cutaneous/tendon xanthomas [1]. Patients with FH cannot effectively clear LDL-C, commonly known as “bad” cholesterol, from the bloodstream. As a result, LDL-C accumulates in the blood, causing early-onset narrowing of the arteries. This predisposes patients to premature atherosclerotic cardiovascular diseases (ASCVDs) [2]. Despite the availability of therapy, FH still remains underdiagnosed and undertreated [3].

FH is caused by mutations in genes encoding the low-density lipoprotein receptor (*LDLR*), apolipoprotein B (*APOB*), and proprotein convertase subtilisin/kexin type 9 (*PCSK9*) and is inherited in an autosomal dominant manner. In cases where the mutation occurs in the low-density lipoprotein receptor adaptor protein 1 (*LDLRAP1*) gene, FH is inherited in an autosomal recessive way. Within all FH-related genes, *LDLR* accounts for the majority (80–85%) of the FH cases, so-called “classic” FH [4,5]. In addition, “nonclassical” FH results from dominantly inherited mutations in the *APOB*, *PCSK9*, and apolipoprotein E (*APOE*) genes, which encode proteins that influence the interaction between the ligands of LDL/LDL-like receptors [6]. FH mutation carriers have a substantially increased risk for CAD in FH. If an individual inherits two copies of the defective gene, one from each parent, the severity of LDL-C levels is significantly increased [5,7].

FH is categorized into heterozygous and homozygous forms based on genotype. The latter represents the most severe form of FH, as LDL-C levels exceeding 13 mmol/L (500 mg/dL) are often linked to a high incidence of early-onset CHD. Currently, homozygous FH (HoFH) patients, with pathogenic mutations in both alleles, usually have plasma LDL-C levels at least twice as high as those in heterozygous FH (HeFH) patients, resulting in levels four times higher than normal [8]. However, a study by Sjouke et al. supposed that only a minority of HoFH patients met the phenotypic diagnostic criteria of >13 mmol/L, and LDL-C (ranges 4.4–21.5 mmol/L) significantly overlapped with levels seen in HeFH patients [4].

Based on recent studies, the estimated occurrence of HeFH in the population is approximately 1 in 250, while the prevalence of HoFH is around 1 in 160,000–300,000, resulting in a much poorer prognosis [9,10]. Cutaneous and tendon xanthomas are often present in the clinical picture of FH. Tendon xanthomas are diagnostic for both HeFH and HoFH, although characteristic skin xanthomas that appear in infancy indicate HoFH [9,11]. Accordingly, young patients with HoFH exhibit more pronounced xanthomas compared to HeFH patients. In cases of neglected treatment, the affected patients have accelerated atherosclerotic CHD from birth, resulting in death from CHD before age 30 [8,12]. Hence, the early clinical and genetic recognition of HoFH is essential for timely treatment to decrease the burden of ASCVDs [13].

In this study, we report the usage of targeted next-generation sequencing (NGS) and bioinformatics analyses to clarify the underlying genetic cause and reach a clinical diagnosis for a suspected case of FH from Kazakhstan.

## 2. Results

### 2.1. Case Presentation

**Figure 1:**
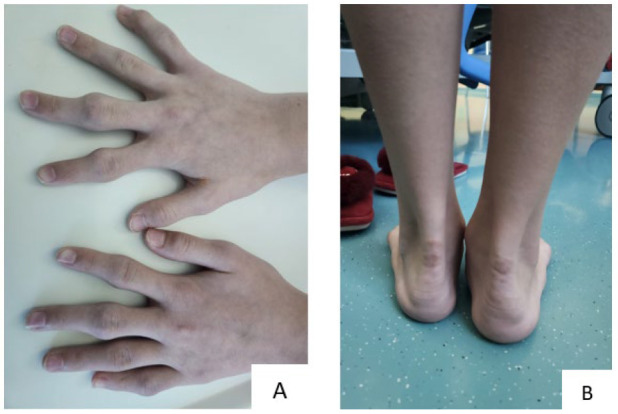
Xanthomas in a 13-year-old girl: (**A**) finger xanthomas, (**B**) Achilles tendon xanthomas.

A 13-year-old Kazakhstani girl presented to the National Research Cardiac Surgery Center (NRCC), Astana, Kazakhstan, with round, protruding lesions on the Achilles tendon and fingers (Figure 1). The patient reported experiencing episodes of syncope and chest pain twice a week. A laboratory examination showed significantly elevated concentrations of total cholesterol (17.96 mmol/L) and LDL-C (16.88 mmol/L), along with normal values of triglycerides (0.038 mmol/L) and high-density lipoprotein cholesterol (HDL-C, 2.71 mmol/L). After examination by a cardiologist, it was confirmed that the lesions were xanthomas, and echocardiography, computed tomography (CT), and Doppler sonography of the extracranial vessels (brachiocephalic trunk) were performed.

According to the ultrasound examination, the results indicated the presence of stenosis and atherosclerosis of the carotid arteries, hemodynamically insignificant stenosis of the lower third of the right common carotid artery (CCA) (40%) and in the middle and lower third of the left CCA (60%), and stenosis of the lumen of the right internal carotid artery (ICA) up to 50% and up to 40% in the left ICA. Also, the left vertebral artery had a small diameter, though the arteries and veins of the lower extremities were passable.

Echocardiography showed a bicuspid aortic valve (BAV) followed by dilation of the ascending aorta. A CT scan of the heart and coronary arteries with contrast also confirmed BAV stenosis, including dilation of the left ventricle.

In addition, the physical examination of the 38-year-old mother and 39-year-old father of the affected patient showed no symptoms of FH, including xanthoma. The parents had no complaints. However, laboratory examination indicated an elevated lipid profile (Table 1).

After the clinical examination, the patient and her parents were sent for further molecular genetic investigation.

### 2.2. Sequencing Analysis

Genetic screening was performed using targeted NGS. The obtained variants were analyzed for protein-coding regions, resulting in the identification of amino acid changes (nonsynonymous), stop loss, stop gain, and frameshift insertion/deletions. The variants were filtered for the quality of the reads. As a result, the affected patient was shown to have 282 variants. For more detailed data, all variants associated with FH genes were selected (Table S1). Notably, the missense nonsynonymous variant c. 295 G>A (p.Glu99Lys) in *LDLR* (rs748944640) was detected in the family trio. According to ClinVar and the ACMG/AMP classifications, c. 295 G>A (p.Glu99Lys) is classified as a pathogenic variant. The p.Glu99Lys pathogenic mutation located in coding exon 3 of the *LDLR* gene results from a G-to-A substitution at nucleotide position 295. This missense variant replaces glutamic acid with lysine at codon 99 of the protein. According to the ExAC database, the minor allele frequency was reported to be 0.00002, while gnomAD showed 0.00001. However, no information was available on the 1000 Genome and Mutation Taster databases.

### 2.3. Validation Sanger Sequencing

**Figure 2:**
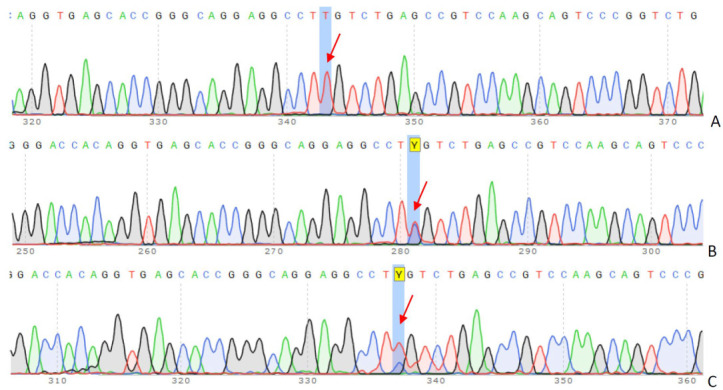
Sanger sequencing and co-segregation analysis in the family trio. The results of c.295 G>A (p.Glu99Lys) mutation in *LDLR*: (**A**) 13-year-old girl (proband), (**B**) father, (**C**) mother. The arrows indicate peaks corresponding to a specific nucleotide (A, T, C, or G) change. Y–nucleotide code, indicating the presence of either C or T.

After the targeted sequencing, the pathogenic genetic variant was confirmed by DNA Sanger sequencing (Figure 2). The genotype of the known variant, c. 295 G>A (p.Glu99Lys), was confirmed to be homozygous in the affected patient. Consequently, the patient’s father and mother are heterozygous carriers of the mutant variant.

The chromatograms above display a sequence of the *LDLR* gene in coding exon 3, revealing a biallelic mutation, c. 295 G>A (p.Glu99Lys), in the proband.

**Figure 3:**
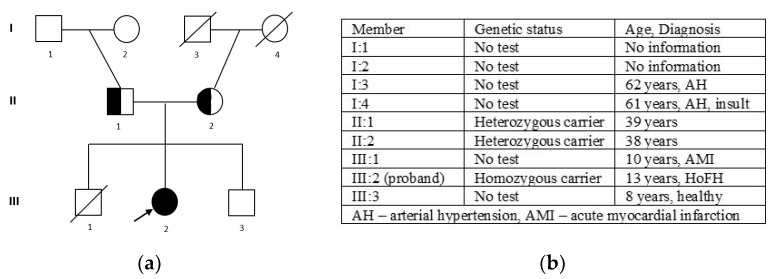
The pedigree of the family (**a**): III 2—proband, homozygous carrier of c. 295 G>A (p.Glu99Lys); II 1—father, heterozygous carrier of c. 295 G>A (p.Glu99Lys); II 2—mother, heterozygous carrier of c. 295 G>A (p.Glu99Lys); and the family members’ phenotypes (**b**).

The patient’s pedigree is demonstrated in Figure 3. The patient with biallelic mutations had homozygous FH.

### 2.4. Genetic Counseling

After the confirmation of HoFH based on the results of the genetic screening, the patient was prescribed LDL-lowering treatment, including LDL apheresis and statins. Further in-depth examination was recommended for the parents of the affected patient. Currently, the family is under observation in the hospital. Additionally, genetic screening is recommended for the patient’s younger brother. Due to the short follow-up time, we have no data in dynamics to show the efficiency of LDL apheresis and statins for a long time period.

## 3. Discussion

In this report, we have described a family case of a 13-year-old child and her parents suspected of a genetic disorder—FH. According to the 2022 Guidelines for Diagnosis and Treatment of Familial Hypercholesterolemia, the diagnostic criteria of HoFH include plasma levels of total cholesterol ≥ 15.52 mmol/L, the presence of xanthomas and atherosclerotic diseases from childhood, and HeFH in parents [2]. If both parents have HeFH, there is a 25% chance that their child will have HoFH and a 50% chance of having a heterozygous FH form [14]. The patient described in our study met several FH indicators, such as early-onset coronary heart disease, an extremely high LDL-C level (16.88 mmol/L) in plasma, and xanthoma formation on both the Achilles tendons and fingers. Even though atherosclerotic symptoms of FH typically appear in adulthood, HoFH patients can experience clinical effects of the disease in the first decades of life, as seen in the 13-year-old patient in our study [8,15]. Moreover, HoFH is much more severe than the heterozygous form of the disorder [9], since the LDL-C level of the affected patient is three times higher than her parents’ levels. HoFH is extremely rare, affecting 1 in 160,000–300,000 [9,10,14]. There is a limited source of literature on pediatric HoFH patients. In the available literature, we found 136 studies, including 45 case reports regarding HoFH in young patients aged 1–12 (children) and 13–17 (adolescents). To our knowledge, this is the first study of its kind not only in Kazakhstan but also across Central Asia. After narrowing our search to a specific mutant variant in the *LDLR* c.418G>A, we found that case reports describing this variant in pediatric patients are exceedingly rare. We identified only two case reports by Chinese scientists, one of which is in Chinese (https://rs.yiigle.com/cmaid/1182389, (accessed on 29 August 2024)). A retrospective cohort study by Tromp et al. presented 751 patients with HoFH from 38 countries, 75% of whom were carriers of biallelic pathogenic variants. Of the patients, 64% were White, 23% were Asian, and the remaining 13% were Black or of mixed race [16]. From childhood, girls with FH have higher total and LDL-cholesterol levels compared to males with FH. LDL-C burden is higher in women with FH at age 30 [17]; therefore, earlier initiation treatment is crucial for reducing lifelong cholesterol.

After sequencing the patient and her unaffected parents, genetic analysis of the annotated variants revealed a disease-causing variant (c.295 G>A p.Glu99Lys) in the *LDLR* gene. Most importantly, a biallelic mutant variant in the *LDLR* gene is known to be homozygous, confirming the HoFH. In our study, the variant was located in coding exon 3 of the *LDLR*, resulting a G-to-A change at nucleotide 295. Glutamic acid at codon 99 was replaced by lysine G, corresponding to transcript variant 3. The alternative variants were described in the research literature and located in exon 4 c.418G>T, c.418G>A p.Glu140Lys. At a molecular level, the variant (known as FH Venezuela/FH Campobasso) that destroys *LDLR* function was first described in 1992 by Hobbs et al. [18]. Several studies described the variant as a pathogenic variant [19,20,21,22,23,24,25]. The variability of known variants is demonstrated in Table 2.

This mutation has been identified in a hypermutable motif, the CpG site, where another four mutations (p.Glu140Asp, p.Glu140*, and p.Glu140Gly) are used to cause the disease [21]. LPL plays a significant role in lipid homeostasis. It is responsible for breaking down triglycerides and phospholipids in plasma lipoproteins through hydrolysis. Moreover, a study on cultured skin fibroblasts from patients with FH shows that the mutation disrupts the function of the *LDLR* protein, resulting in elevated cholesterol levels in the bloodstream [26,27].

A recent study in Japan reported more than 4970 variants of the *LDLR* gene in 650 unrelated FH patients. Based on the results of this study, the proportion of *LDLR* pathogenic variants was more significant in patients who developed CAD at a younger age, and it significantly declined as the age of CAD onset increased. Also, the frequency of the known variant (c.418G>A) was only 1.4% [23]. Another Italian study on children (n = 264) with FH showed that individuals carrying *LDLR*-negative mutations, which completely eliminate *LDLR* function, had a more severe phenotype, particularly elevated plasma lipid levels and greater aorta/carotid intima–media thickness [28]. A recent study on a Chinese 9-year-old boy with HoFH detected the same mutation in the *LDLR*: c.418G>A (p.E140K) inherited from both parents (heterozygous carriers) as well as in our case. His phenotypic presentation showed multiple xanthomas and thickening of the carotid arteries, although no structural abnormalities were observed in the heart [25]. The molecular genetic identification of mutant *LDLR* alleles is necessary for a definitive diagnosis of FH. In addition, it enables a 50% success rate in screening the first-degree relatives of affected individuals, facilitating the early diagnosis and prevention of ASCVDs [29]. Most (90%) of HoFH patients have pathogenic variants in both alleles of the *LDLR* [19].

Due to mutations in two alleles, HoFH has a more expressed lethal effect; children ≤ 4 years of age have experienced sudden death due to acute myocardial infarction (AMI). In the family history, the patient’s older sibling died from AMI at the age of 10. The presence of BAV stenosis in our patient is life-threatening; therefore, aortic valve replacement is required for young adults with HoFH [30].

In our research, genetic analysis also revealed other FH-related variants in the genes *APOE*, *APOB*, *PCSK9*, and *LDLRAP1*. However, the pathogenicity of the observed variants rejected disease-causing patterns. According to the ACMG/AMP criteria, the annotated variants were classified as benign/likely benign, showing no effect on the disease. At the same time, the pathogenicity of the *APOB* (p.D2527H) gene was still undefined (Table S1). Therefore, variants of uncertain significance (VUSs) have insufficient clinical information and require further functional studies.

LDL-C lowering by statins, PCSK9 inhibitors, and lipoprotein apheresis can reduce xanthomas in patients with FH [31]. A systematic review of 76 case reports on HoFH in children found that lipoprotein apheresis is a generally safe method that facilitates LDL-C lowering and reducing xanthoma growth; however, its efficacy in preventing CVD is understudied [32]. A previously mentioned study by Chinese scientists showed that lipoprotein apheresis therapy combined with atorvastatin significantly reduced LDL-C levels [25]. Another study on a 10-year-old patient revealed positive results when treatment was held collectively with statins and lipoprotein apheresis [33]. Japanese research on drug therapy with probucol demonstrated an LDL-C-lowering effect in HoFH patients, which can cause a reduction in xanthomas on the cutaneous and Achilles tendons [2]. A recent study by Wiegman et al. [34] presented evinacumab, a novel LDLR-independent lipid-lowering therapy, which has proven effective in reducing LDL-C levels by nearly 50% in high-risk pediatric HoFH patients, demonstrating both its efficacy and safety. The guidelines for managing HoFH in pediatric patients recommend starting LDL apheresis treatment for children with HoFH as early as possible [14]. Moreover, early LDL-lowering treatment could be effective in the regression of xanthomas and lowering the risk of cardiovascular events. Despite the minimal effect on hypercholesterolemia, patients with HoFH should follow a heart-healthy, low-saturated fat, and low-cholesterol diet. Concretely, saturated fatty acids should account for <7% of energy, and cholesterol intake should be limited to 200 mg/day [14]. Also, the prevention of smoking and obesity control are essential for the treatment. Regular physical activity is encouraged, but aortic involvement must be assessed before performing sports [2,8]. In addition, cascade screening should be performed for untested relatives to assess and mitigate CVD risks.

Tromp et al. suggested that the HoFH guidelines for screening and treatment have mainly relied on studies conducted among patients of European ancestry or developed countries, highlighting that outcomes are linked to untreated LDL-C levels, genetic mutations, and the age of treatment initiation. Patients from low-income countries often present with more severe symptoms at diagnosis, have limited access to advanced treatments, and experience cardiovascular events about a decade earlier than patients in wealthier countries [16]. Therefore, our research is particularly valuable for developing Central Asian countries, including Kazakhstan, where relevant data are essential for clinicians.

Our study successfully identified a disease-causing variant in the patient through genetic screening (targeted NGS), thereby substantiating the diagnosis of HoFH. Based on these findings, the patient was immediately referred for appropriate treatment involving statins and LDL apheresis, as HoFH is a life-threatening disorder that strongly requires early treatment. Globally, patients with HoFH are often diagnosed too late and receive insufficient treatment [3]. Research by Japanese scientists has shown that the average life expectancy of HoFH patients increased from 28 to 59 years with statin treatment [2]. Overall, this case report emphasizes the importance of strengthening clinical awareness for HoFH among cardiologists and healthcare providers worldwide, highlighting the potential for early diagnosis and intervention in managing this rare genetic disorder in pediatric patients.

This case report has some limitations. Firstly, it focuses on a single case. Due to the rareness of the disorder, it establishes a limited source of literature in this field. Generally, the lack of data on the Kazakh population is challenging, as ethnic Kazakhs have mixed heterogeneous ancestry. The genetic screening was performed only for the family trio. A molecular genetic investigation is recommended for untested family members, especially the proband’s younger brother. Lastly, the short follow-up period prevents us from assessing the long-term efficacy of LDL apheresis and statin therapy.

In summary, this study reported a young patient diagnosed with HoFH caused by a pathogenic variant in the *LDLR* gene. This variant is located in coding exon 3 of the *LDLR*, resulting in a G-to-A change at nucleotide 295, where glutamic acid is replaced by lysine—transcript variant 3. To our knowledge, this is the first study of its kind to be reported in Central Asia. It is well known that HoFH is a severe and rare genetic disorder that can progressively lead to life-threatening cardiac consequences if timely therapy is neglected. After confirming the genetic diagnosis of HoFH, the patient received intensive LDL-lowering treatments in a timely manner, including lipoprotein apheresis and statin administration. Given the life-threatening, underdiagnosed, and undertreated nature of HoFH worldwide, this case highlights the need for increased awareness among healthcare providers, as early detection and intervention are important for improving outcomes, particularly in pediatric patients with this rare genetic disorder.

## 4. Materials and Methods

This study was approved by both Ethics Committees at National Laboratory Astana, Nazarbayev University (No. 02-2023 from 1 April 2023), and at the NRCC, Astana, Kazakhstan (No. 16 from 24 April 2023). The study was conducted in accordance with the Declaration of Helsinki. Informed consent for genetic analysis was obtained from the patient and her parents. A family trio, including the affected 13-year-old girl and unaffected parents, presented to the case report. Clinical and epidemiological data were collected from patients’ medical records by cardiologists at the NRCC.

Venous blood samples were collected from the patient and her parents. Genomic DNA (gDNA) was extracted from 200 µL of venous blood sample using a QIAamp DNA Mini Kit: DNA purification from Blood or Body Fluids (Qiagen, Hilden, Germany) according to the manufacturer’s instructions. The concentration and purity of gDNA were determined by measuring absorbance at 260 nm using a NanoDrop™ Spectrophotometer (ThermoFisher Scientific, Waltham, MA, USA). The quantity of extracted DNA was determined by Qubit 2000 (ThermoFisher Scientific, Waltham, MA, USA).

Targeted sequencing was performed to identify genetic variants contributing to FH. DNA libraries were prepared according to the standard protocol “Illumina DNA Prep with Enrichment” provided by Illumina, Inc. and sequenced on a Novaseq 6000 platform (Illumina Inc., San Diego, CA, USA). An Illumina TruSight cardio panel (Illumina Inc., Santa Clara, CA, USA) was used for genetic screening. The cardio panel consists of 174 genes associated with cardiac disorders, including FH. The panel includes several genes associated with FH: *LDLR*, *PCSK9*, *LDLRAP1*, *APOB*, *APOE*, *APOA4*, *APOA5*, *ABCG5*, *LPL*, etc.

All sequencing reads were aligned to the human genome reference build GRCh37 (hg19) using the bwa (v0.7.17) tool, followed by variant calling and annotation. Single-nucleotide variants (SNVs) and insertion/deletions (indels) were detected using the GATK3 (v.3.8-1-0-gf15c1c3ef). SNVs located in the intron region and synonymous variants that did not affect the splicing site were excluded. The ANNOVAR (v20190101) tool was used to annotate the genetic variants for location, corresponding genes, and transcript length. The GATK3 workflow was used to analyze and annotate the raw data, and VCF files were generated. Then, the variants were filtered for a quality ≥ 20.

The clinical significance of the genetic variants was interpreted by the guidelines developed by the American College of Medical Genetics and Genomics (ACMG) and the Association of Molecular Pathology (AMP) in 2015. Overall, genetic variants were classified into five categories of ACMG/AMP standards: pathogenic (P), likely pathogenic (LP), a variant of uncertain significance (VUS), likely benign (LB), or benign (B) [35,36]. Variant interpretations were made on the InterVar (https://wintervar.wglab.org/ (accessed on 5 August 2024) and ClinVar (https://www.ncbi.nlm.nih.gov/clinvar/) (accessed on 5 August 2024) platforms. In addition, annotated variants were compared with international genomic databases the Exome Aggregation Consortium (ExAC), the 1000 Genome (1000G), the Genome Aggregation Database (gnomeAD), Online Mendelian Inheritance in Man (OMIM), Mutation Taster, ESP, NCBI, etc.

To confirm potential pathogenic variants identified by targeted sequencing, DNA Sanger sequencing was performed on gDNA obtained from the blood samples of family members. Accordingly, forward and reverse primers were designed using the DNA-oligonucleotide synthesizer ASM-800 (Biosset, Novosibirsk, Russia). Information about all primers for polymerase chain reaction (PCR) is shown in Table 3.

Amplified PCR was performed in a final volume of 20 µL, containing 50 ng/µL of the gDNA, 10 pmol of the forward and reverse primers (LDLR_F and LDLR_R), 4 µL of 5× Buffer 2.5 mM dNTP (Fermentas, Lithuania, Vilnius), and 0.2 U of Phusion™ High-Fidelity DNA Polymerase (ThermoFisher, Cleveland, OH, USA). The thermal cycling conditions for PCR included initial denaturation for 5 min at 96 °C, followed by 35 cycles of denaturation at 94 °C for 30 s, annealing at 58 °C for 45 s, elongation at 72 °C for 45 s, and a final extension for 10 min at 72 °C. The PCR products were run on 1% agarose gel to detect DNA amplicon size. After that, PCR products were purified using the ExoSAP-IT Express PCR Product Cleanup (Thermo Fisher Scientific, Wilmington, Germany). DNA sequencing of the obtained PCR products was carried out with the BigDye Terminator Cycle Sequencing v.3.1 kit (Applied Biosystems, Foster City, CA, USA). Finally, sequencing analysis was conducted using the Genetic DNA Analyzer (Applied Biosystems, Foster City, CA, USA).

## Figures and Tables

**Table 1:** Lipid profile of the family trio.

Subject	Proband	Father	Mother
Cholesterol, mmol/L	17.96 ↑	6.82 ↑	6.39 ↑
ApoA I, mmol/L	25.9 **↓**	55.5	43.9
Triglyceride, mmol/L	0.038	0.98	0.77
LDL-C, mmol/L	16.88 ↑	5.32 ↑	4.93 ↑
HDL-C, mmol/L	2.71	1.47	1.31 **↓**
ApoB, umol/L	8.90 ↑	2.85	2.32
Lp(a), nmol/L	133.6 ↑	10.5	35.5

ApoA I—apolipoprotein A I, ApoB—apolipoprotein B, Lp(a)—lipoprotein A, ↑—increased level, **↓**—decreased level.

**Table 2:** Variability of known variants in *LDLR* gene in studies.

Gene	Nucleotide Change	Protein Change	Study	Number of Affected Patients	Reference
* LDLR *	c.418G>T	p.Glu140 *	Cohort study (n = 4703)	2	Do R et al. [19], 2015
* LDLR *	c.418G>A	p.E140K	Cohort study (n = 136)	1	Han et al. [20], 2015
* LDLR *	c.418G>A	p.Glu140Lys	Family study (n = 11)	1	Hernández et al. [21], 2018
* LDLR *	c.418G>T	p.Glu140 *	Cohort study (n = 127)	2	Di Taranto et al. [22], 2019
* LDLR *	c.418G>A	p.Glu140Lys	Cohort study (n = 650)	6	Hori et al. [23], 2019
* LDLR *	c.418G>T	p.(E140 *)	Cohort study (n = 125)	1	Bertolini et al. [24], 2020
* LDLR *	c.418G>A	p.Glu140Lys	Case report	1	Xu et al. [25], 2022

* premature stop codon.

**Table 3:** Primer sequences and characteristics.

Primer	Sequence (5′–3′)	T_a_ (°C)	Amplicon Length (bp)
* LDLR_F *	AGACTTCACACGGTGATGGT	58 °C	529
* LDLR_R *	TTGGAAATCCACTTCGGCAC	58 °C	529

*LDLR*, low-density lipoprotein receptor; T_a_, annealing temperature; F, forward; R, reverse.

## Data Availability

Please contact National Laboratory Astana (via phone or mail) for researchers who meet the criteria for access to confidential data. The data underlying the results presented in the study are available from the authors: phone number: +7-7172706501, mail: akilzhanova@nu.edu.kz.

## Supplementary Materials

The following supporting information can be downloaded at: https://www.mdpi.com/article/10.3390/ijms252211882/s1.
