# Supplementary material for: Targeted NGS Revealed Pathogenic Mutation in a 13-Year-Old Patient with Homozygous Familial Hypercholesterolemia: A Case Report
Source: Int J Mol Sci. 2024 Nov 5;25(22):11882. doi: 10.3390/ijms252211882 (PMC11593385; doi:10.3390/ijms252211882)
Supplement: Supplementary file 1 [file ijms-25-11882-s001.zip › ijms-3280435-supplementary.pdf]

Table S1. List of genetic variants related to FH

Sample	Gene	Position	Transcript	Exonic effect	Genotype	OMIM	1000G	ExAC	Mutation Taster	gnomAD	Rs ID	ClinVar	ACMG/AMP classification	Phenotype
FH001	*LDLR*	11216000	NM_001195799:exon3 c.G295A:p.E99K	nonsynonymous SNV	**homozygous**	606945	n/a	0.00002	1.0	n/a	rs748944640	**Pathogenic/ Likely_pathogenic**	**Pathogenic**	Familial_hypercholesterolemia
FH001	*APOE*	45412080	NM_000041:exon4 c.C526T:p.R176C	nonsynonymous SNV	heterozygous	107742	0.075	0.072	0.930	0.0833	rs7412	Benign	Benign	Hyperlipoproteinemia, type III
FH001	*PCSK9*	55529187	NM_174936:exon12:c.G2009A:p.G670E	nonsynonymous SNV	heterozygous	607786	0.9	0.94	1.0	0.8930	rs505151	Benign/Likely benign	Benign	Familial_hypercholesterolemia| Familial_hypobetalipoproteinemia| Hypercholesterolemia,_autosomal_ dominant,_3
FH001	*PCSK9*	55524237	NM_174936:exon9:c.G1420A:p.V474I	nonsynonymous SNV	homozygous	607786	0.87	0.85	1.0	0.8315	rs562556	Benign	Benign	Familial_hypercholesterolemia| Familial_hypobetalipoproteinemia| Hypercholesterolemia,_autosomal_ dominant,_3
FH001	*LDLRAP1*	25889632	NM_015627:exon6:c.T604C:p.S202P	nonsynonymous SNV	heterozygous	605747	0.43	0.5	1.0	0.4730	rs6687605	Benign/Likely benign	Benign	Familial_hypercholesterolemia|
FH001	*APOB*	21225281	NM_000384:exon29:c.G13013A:p.S4338 N	nonsynonymous SNV	heterozygous	107730	0.63	0.71	1.0	0.7647	rs1042034	Benign/Likely benign	Benign	Familial_hypercholesterolemia| Familial_hypobetalipoproteinemia
FH001	*APOB*	21232161	NM_000384:exon26:c.G7579C:p.D2527H	nonsynonymous SNV	heterozygous	107730	n/a	n/a	0.999	n/a	n/a	n/a	Uncertain Significance	n/a
FH001	*APOB*	21232803	NM_000384:exon26:c.A6937G:p.I2313V	nonsynonymous SNV	homozygous	107730	0.99	0.99	1.0	0.9842	rs584542	n/a	Benign	n/a
FH001	*APOB*	21235475	NM_000384:exon26:c.A4265G:p.Y1422C	nonsynonymous SNV	homozygous	107730	1.0	1.0	n/a	0.9997	rs568413	n/a	Benign	n/a
FH001	*APOB*	21231524	NM_000384:exon26:c.C8216T:p.P2739L	nonsynonymous SNV	heterozygous	107730	0.37	0.29	0.000	0.2347	rs676210	Benign/Likely benign	Benign	Familial_hypercholesterolemia| Familial_hypobetalipoproteinemia
FH002	*LDLR*	11216000	NM_001195799:exon3 c.G295A:p.E99K	nonsynonymous SNV	**heterozygous**	606945	n/a	0.00002	1.0	n/a	rs748944640	**Pathogenic/ Likely_pathogenic**	**Pathogenic**	Familial_hypercholesterolemia
FH002	*APOE*	45412080	NM_000041:exon4 c.C526T:p.R176C	nonsynonymous SNV	heterozygous	107742	0.075	0.072	0.930	0.0833	rs7412	Benign	Benign	Hyperlipoproteinemia, type III
FH002	*PCSK9*	55529187	NM_174936:exon12:c.G2009A:p.G670E	nonsynonymous SNV	heterozygous	607786	0.9	0.94	1.0	0.8930	rs505151	Benign/Likely benign	Benign	Familial_hypercholesterolemia| Familial_hypobetalipoproteinemia| Hypercholesterolemia,_autosomal_ dominant,_3
FH002	*PCSK9*	55524237	NM_174936:exon9:c.G1420A:p.V474I	nonsynonymous SNV	homozygous	607786	0.87	0.85	1.0	0.8315	rs562556	Benign	Benign	Familial_hypercholesterolemia| Familial_hypobetalipoproteinemia| Hypercholesterolemia,_autosomal_ dominant,_3
FH002	*LDLRAP1*	25889632	NM_015627:exon6:c.T604C:p.S202P	nonsynonymous SNV	homozygous	605747	0.43	0.5	1.0	0.4730	rs6687605	Benign/Likely benign	Benign	Familial_hypercholesterolemia|
FH002	*APOB*	21225281	NM_000384:exon29:c.G13013A:p.S4338 N	nonsynonymous SNV	homozygous	107730	0.63	0.71	1.0	0.7647	rs1042034	Benign/Likely benign	Benign	Familial_hypercholesterolemia| Familial_hypobetalipoproteinemia
FH002	*APOB*	21232161	NM_000384:exon26:c.G7579C:p.D2527H	nonsynonymous SNV	heterozygous	107730	n/a	n/a	0.999	n/a	n/a	n/a	Uncertain Significance	n/a
FH002	*APOB*	21232803	NM_000384:exon26:c.A6937G:p.I2313V	nonsynonymous SNV	homozygous	107730	0.99	0.99	1.0	0.9842	rs584542	n/a	Benign	n/a
FH002	*APOB*	21235475	NM_000384:exon26:c.A4265G:p.Y1422C	nonsynonymous SNV	homozygous	107730	1.0	1.0	n/a	0.9997	rs568413	n/a	Benign	n/a
FH002	*APOB*	21250914	NM_000384:exon14:c.C1853T:p.A618V	nonsynonymous SNV	heterozygous	107730	0.48	0.49	0.000	0.4190		Benign/Likely benign	Benign	Familial_hypercholesterolemia| Familial_hypobetalipoproteinemia
FH003	*LDLR*	11216000	NM_001195799:exon3 c.G295A:p.E99K	nonsynonymous SNV	**heterozygous**	606945	n/a	0.00002	1.0	n/a	rs748944640	**Pathogenic/ Likely_pathogenic**	**Pathogenic**	Familial_hypercholesterolemia

Sample	Gene	Position	Transcript	Exonic effect	Genotype	OMIM	1000G	ExAC	Mutation Taster	gnomAD	Rs ID	ClinVar	ACMG/AMP classification	Phenotype
FH003	*APOE*	45412080	NM_000041:exon4 c.C526T:p.R176C	nonsynonymous SNV	heterozygous	107742	0.075	0.072	0.930	0.0833	rs7412	Benign	Benign	Hyperlipoproteinemia, type III
FH003	*PCSK9*	55529187	NM_174936:exon12:c.G2009A:p.G670E	nonsynonymous SNV	heterozygous	607786	0.9	0.94	1.0	0.8930	rs505151	Benign/Likely benign	Benign	Familial_hypercholesterolemia|Familial_hypobetalipoproteinemia|Hypercholesterolemia,_autosomal_dominant,_3
FH003	*PCSK9*	55524237	NM_174936:exon9:c.G1420A:p.V474I	nonsynonymous SNV	homozygous	607786	0.87	0.85	1.0	0.8315	rs562556	Benign	Benign	Familial_hypercholesterolemia|Familial_hypobetalipoproteinemia|Hypercholesterolemia,_autosomal_dominant,_3
FH003	*LDLRAP1*	25889632	NM_015627:exon6:c.T604C:p.S202P	nonsynonymous SNV	heterozygous	605747	0.43	0.5	1.0	0.4730	rs6687605	Benign/Likely benign	Benign	Familial_hypercholesterolemia|
FH003	*APOB*	21231524	NM_000384:exon26:c.C8216T:p.P2739L	nonsynonymous SNV	heterozygous	107730	0.37	0.29	0.000	0.2347	rs676210	Benign/Likely benign	Benign	Familial_hypercholesterolemia|Familial_hypobetalipoproteinemia
FH003	*APOB*	21250914	NM_000384:exon14:c.C1853T:p.A618V	nonsynonymous SNV	heterozygous	107730	0.48	0.49	0.000	0.4190	rs679899	Benign/Likely benign	Benign	Familial_hypercholesterolemia|Familial_hypobetalipoproteinemia
FH003	*APOB*	21224853	NM_000384:exon26:c.G13441A:p.A4481T	nonsynonymous SNV	heterozygous	107730	0.014	0.024	1.0	0.0210	rs1801695	Conflicting	Benign	Familial_hypercholesterolemia|Hypercholesterolemia,_autosomal_dominant,_type_B
FH003	*APOB*	21255373	NM_000384:exon10:c.A1205G:p.H402R	nonsynonymous SNV	heterozygous	107730	n/a	n/a	1.0	n/a	n/a	n/a	Uncertain Significance	n/a
FH003	*APOB*	21232803	NM_000384:exon26:c.A6937G:p.I2313V	nonsynonymous SNV	homozygous	107730	0.99	0.99	1.0	0.9842	rs584542	n/a	Benign	n/a
FH003	*APOB*	21235475	NM_000384:exon26:c.A4265G:p.Y1422C	nonsynonymous SNV	heterozygous	107730	1.0	1.0	n/a	0.9997	rs568413	n/a	Benign	n/a
FH003	*APOB*	21231524	NM_000384:exon26:c.C8216T:p.P2739L	nonsynonymous SNV	heterozygous	107730	0.37	0.29	0.000	0.2347	rs676210	Benign/Likely benign	Benign	Familial_hypercholesterolemia|Familial_hypobetalipoproteinemia

*FH001 - affected patient (proband), FH002 - father, FH003-mother
